# Altered monetary loss processing and reinforcement-based learning in individuals with obesity

**DOI:** 10.1007/s11682-017-9786-8

**Published:** 2017-12-29

**Authors:** Jana Kube, David Mathar, Annette Horstmann, Sonja A. Kotz, Arno Villringer, Jane Neumann

**Affiliations:** 1 Max Planck Institute for Human Cognitive and Brain Sciences, Stephanstraße 1a, 04103 Leipzig, Germany; 2 IFB Adiposity Diseases, Leipzig University Medical Center, Leipzig, Germany; 3 Faculty 5 - Business, Law and Social Sciences, Brandenburg University of Technology Cottbus-Senftenberg, Cottbus, Germany; 4 Department of Psychology, University of Cologne, Cologne, Germany; 5 Department of Neuropsychology and Psychopharmacology, Faculty of Psychology and Neuroscience, Maastricht University, Maastricht, Netherlands; 6 Clinic of Cognitive Neurology, University Hospital Leipzig, Leipzig, Germany; 7 Mind & Brain Institute, Berlin School of Mind and Brain, Humboldt-University, Berlin, Germany; 8 Department of Medical Engineering and Biotechnology, University of Applied Sciences, Jena, Germany

**Keywords:** Fmri, Obesity, Prediction error, Reward, Reinforcement, Money

## Abstract

Individuals with obesity are often characterized by alterations in reward processing. This may affect how new information is used to update stimulus values during reinforcement-based learning. Here, we investigated obesity-related changes in non-food reinforcement processing, their impact on learning performance as well as the neural underpinnings of reinforcement-based learning in obesity. Nineteen individuals with obesity (BMI > = 30 kg/m^2^, 10 female) and 23 lean control participants (BMI 18.5–24.9 kg/m^2^, 11 female) performed a probabilistic learning task during functional magnetic resonance imaging (fMRI), in which they learned to choose between advantageous and disadvantageous choice options in separate monetary gain, loss, and neutral conditions. During learning individuals with obesity made a significantly lower number of correct choices and accumulated a significantly lower overall monetary outcome than lean control participants. FMRI analyses revealed aberrant medial prefrontal cortex responses to monetary losses in individuals with obesity. There were no significant group differences in the regional representation of prediction errors. However, we found evidence for increased functional connectivity between the ventral striatum and insula in individuals with obesity. The present results suggest that obesity is associated with aberrant value representations for monetary losses, alterations in functional connectivity during the processing of learning outcomes, as well as a decresased reinforcement-based learning performance. This may affect how new information is incorporated to adjust dysfunctional behavior and could be a factor contributing to the maintenance of dysfunctional eating behavior in obesity.

**Electronic supplementary material:**

The online version of this article (10.1007/s11682-017-9786-8) contains supplementary material, which is available to authorized users.

Previous studies have reported obesity-related alterations in the neural representation of rewarding food stimuli (Feldstein Ewing et al. 2016; Stice et al. 2009; García-García et al. 2014). However, while the processing of food reward has been studied extensively in obesity, non-food reward likewise provides a powerful source of information to monitor and successfully adapt behavior to changing environments. In this vein, Saunders and Robinson (2013) hypothesized that humans generally differ in their reward cue reactivity, a trait that is likely to be stable across different domains of primary and secondary reinforcers. Indeed, obesity-related alterations in reward processing have recently been shown to exist outside of the food context. For instance, Opel et al. (2015) reported increased neural responses of individuals with obesity in areas of the brain’s reward circuit following the presentation of monetary gains. While these authors found no significant group differences during the processing of monetary losses, Balodis et al. (2013) described that individuals with obesity exhibited greater neural activation in subcortical as well as prefrontal brain areas during the anticipation of monetary gains and losses, suggesting that obesity-related alterations in reinforcement processing may also exist for aversive stimuli.

Interestingly, Balodis et al. (2013) additionally found a dissociation between neural responses during the anticipation and receipt of monetary reinforcement, a phenomenon that has similarly been observed in the food context. Specifically, individuals with obesity tend to show increased neural responses during the anticipation (Rothemund et al. 2007), but blunted responses during the actual receipt of rewarding food stimuli (Stice et al. 2008, 2010). Prominent theories argue that this discrepancy results from an initially high trait reward responsiveness facilitating overeating and subsequent neuroadaptive processes, leading to a heightened motivational value of anticipated food, but blunted hedonic signals when actually consuming it (Kenny 2011). Others argue that both a high and low reward responsiveness may be associated with obesity (Val-Laillet et al. 2015). Importantly, Kroemer and Small (2016) suggest that the apparent dissociation between responses during the anticipation and receipt of rewarding (food) stimuli may instead be explained in terms of altered reinforcement-based learning in individuals with obesity. Specifically, individuals with obesity displayed heightened reward sensitivity, but lower learning rates leading to increased neural responses during the anticipation, but blunted striatal responding during the receipt of rewarding (food) stimuli.

While animal studies have, indeed, shown that obesity is also associated with alterations in learning and behavioral adaptation (Reichelt et al. 2014; Johnson and Kenny 2010; Kanoski and Davidson 2011), few studies have investigated reinforcement-based learning in humans with obesity. Using the Iowa Gambling Task, Horstmann et al. (2011) demonstrated that women with obesity in contrast to lean women preferred choice options associated with high immediate monetary rewards even in light of high potential losses, and failed to adjust their behavior over time despite an overall negative outcome. Recently, Coppin et al. (2014) reported evidence suggesting that these deficits may be driven by impaired reinforcement-based learning. Using two different tasks, they found that individuals with obesity failed to develop a preference for the most rewarded patterns in a cue conditioning paradigm, and also showed less avoidance for negative stimuli in a probabilistic learning task. Interestingly, performance was partly affected by working memory differences between lean participants and participants with obesity. Together, these studies suggest that obesity may be associated with alterations in neural reinforcement processing beyond the food context that may also affect decision making and reinforcement-based learning.

Electrophysiological and neuroimaging studies in normal-weight populations highlight the role of a dopaminergic prediction error (PE) signal for learning and updating stimulus and action values when a presented outcome is better or worse than expected (Schultz et al. 1997; Garrison et al. 2013; Chase et al. 2015). Alterations in the coding of dopaminergic PEs in the striatum as well as the transfer of feedback signals to higher cortical areas have been found to be associated with a reduced learning performance. For instance, successful learners exhibit more robust PE signals in the dorsal and ventral striatum (VS) than less successful ones (Schönberg et al. 2007), while a decline in learning performance with age seems to be related to a reduction in PE-related blood oxygenation level dependent (BOLD) activity in the VS (Eppinger et al. 2013). Moreover, Park et al. (2010) reported that individuals with alcohol dependence show a reduced learning performance despite intact ventral striatal PE-responses, which was, however, associated to alterations in the functional connectivity between the VS and dorsolateral prefrontal cortex. Accordingly, it seems that both PE coding in the VS and its functional utilization in other brain areas may be potential mechanisms that evoke impaired decision making and learning. Indeed, individuals with obesity have been shown to have an altered dopaminergic circuitry, such as a lower striatal D2-receptor binding potential (Wang et al. 2001). This further highlights the possibility that alterations in neural PE signaling may affect feedback utilization in reinforcement-based learning in individuals with obesity.

In the current study we used functional magnetic resonance imaging (fMRI) to investigate the neural mechanisms of monetary gain and loss processing and the neural underpinnings of feedback utilization in reinforcement-based learning in individuals with obesity. We aimed to (1) further examine whether individuals with obesity are characterized by alterations in reinforcement processing beyond the food context; (2) test whether these alterations affect the neural representation of both monetary gains and losses as well as their omission and avoidance; (3) replicate previous findings regarding obesity-related alterations in learning performance and examine whether learning deficits are present for both performance in learning from reward and performance in learning from punishment, and (4) investigate the neural correlates of reinforcement-based learning, specifically the representation and utilization of PE signals in the brain.

We hypothesized that individuals with obesity show altered neural representations of both positive and negative monetary outcomes in areas of the brain’s reward system, such as the striatum, medial orbitofrontal cortex (OFC), insula, midbrain and thalamus. Further, we hypothesized that individuals with obesity would exhibit a lower reinforcement-based learning performance, which potentially is mediated by alterations in ventral striatal PE processing.

## Materials and methods

### Participants

Fifty-five participants were recruited for the current study via online advertisements, and from the participant database of the Max Planck Institute for Human Cognitive and Brain Sciences, Leipzig, Germany. Inclusion criteria encompassed MR-eligibility, right-handedness, an age-range of 20 to 45 years, as well as a BMI between 30.0 and 50.0 kg/m^2^ for participants with obesity, or a BMI between 18.5 and 24.9 kg/m^2^ for lean control participants. Participants were excluded from the study if they reported current smoking, the use of drugs or psychoactive medication, a history of neuropsychiatric diseases, current depressive symptoms (Beck’s Depression Inventory, BDI-SF, > = 10, Beck and Steer 1987), or a thyroid disease. We restricted our sample by these criteria to avoid confounding influences of age (e.g. Samanez-Larkin et al. 2012), smoking status (e.g. Martin et al. 2014), as well as neuropsychiatric symptoms and medication (e.g. Etkin and Wager 2007; Philip et al. 2012; Zhang et al. 2013; Wittmann and D’Esposito 2015; Yan et al. 2015) on reinforcement processing. Furthermore, participants reporting thyroid diseases were excluded from the current sample as these conditions may affect body-weight status (Tzotzas et al. 2000).

Upon participation we had to exclude two participants due to lack of task compliance (one lean, one obese), and three participants due to lack of task comprehension (one lean, two obese). Four participants were excluded who reported obesity at the time of recruitment, but fell below our predefined BMI criteria for obesity at the time of measurement, and three participants were excluded due to current depressive symptomatology (BDI-SF > = 10; one lean, one obese) or medication use (one obese). Finally, one participant experienced a panic attack inside the scanner and aborted the scanning session.

The final sample thus consisted of 19 individuals with obesity and 23 individuals without obesity who were comparable with respect to gender, age, education, and working memory performance (Table 1).

**Table 1:** Sample characteristics

	Participants with obesity *n* = 19	Participants without obesity *n* = 23	Test statistic
Demographics			
Female/male	10/9	11/12	***Χ***^2^ = 0.096, *p* = .757
Age	29.5 ± 5.6	30.0 ± 5.0	t(40) = − 0.264, *p* = .793
Years of education	11.8 ± 0.7	11.9 ± 0.4	U = 216.000, *p* = .864
Income	6/6/5/0/1	5/9/6/1/1	***Χ***^2^ = 1.996, *p* = .976
Occupation	1/2/9/6/1	0/0/10/9/4	***Χ***^2^ = 4.541, *p* = .299
Anthropometrics			
BMI	35.4 ± 4.5	22.4 ± 1.7	U = 0.000, *p* < .001
WHR	0.9 ± 0.1	0.7 ± 0.2	U = 78.500, *p* < .001
Weight duration	15.07 ± 7.24	– ^1^	
Tests and questionnaires			
WMS-R FM	8.3 ± 1.2	8.8 ± 1.0	U = 167.500, *p* = .172
BDI-SF	2.6 ± 2.6	1.3 ± 1.5	U = 158.000, *p* = .107
BIS/BAS-BIS	2.6 ± 0.6	2.9 ± 0.4	t(40) = − 1.930, *p* = .061
BIS/BAS-BAS	3.1 ± 0.3	3.1 ± 0.3	t(40) = 0.167, *p* = .868

Years of education = years of school education, Income = Available income measured in five categories (< 500€ per month, 500–1000€ per month, 1000–1500€ per month, 1500–2000€ per month, > 2000€ per month), data available from *n* = 22 lean and *n* = 18 obese participants, Occupation = current occupation classified according to five categories (unemployed, trainee, student, employee, self-employed), BMI = Body Mass Index, WHR = Waist-to-hip-ratio, Weight duration = average number of years participants with obesity had been obese. ^1^All but one lean participant reported they had been lean throughout their lives. WMS-R FM – Wechsler Memory Scale - Revised, Subtest Figural Memory, BDI-SF = Beck’s Depression Inventory – Short Form, BIS/BAS - BIS = Behavioral Inhibition/Behavioral Activation Scale – Subscale Behavioral Inhibition, BIS/BAS - BAS = Behavioral Inhibition/Behavioral Activation Scale – Subscale Behavioral Activation. Values represent mean ± SD. Independent samples t-tests are reported for normally distributed data, while results of the Mann–Whitney U-test are reported if the assumption of normality was violated (as determined by Shapiro–Wilk test). ***Χ***^2^ and results from Fisher’s Exact test are reported for comparisons of categorical data

All participants gave written informed consent prior to their participation and received 8 €/hour for reimbursement (mean study duration 2 h). Additionally, participants received a monetary bonus dependent on their performance in the reinforcement learning task (final score/1000, on average 3.10 €). The study was carried out in accordance with the Declaration of Helsinki and was approved by the ethics committee of the University of Leipzig.

### Procedure and probabilistic reinforcement learning task

Participants performed a probabilistic reinforcement learning task comprising 240 trials, which we adapted from Kim et al. (2006). In each trial participants were presented with a pair of symbols and had to choose one of them by button press. Three types of pairs were included in the experiment: (1) one pair signaled the possibility of winning 50 points or receiving no outcome (gain condition, 80 trials), (2) one pair signaled the possibility of losing 50 points or receiving no outcome (loss condition, 80 trials), and (3) one pair was associated with a neutral outcome signaling neither gain nor loss (neutral condition, 80 trials). In each pair of stimuli one symbol had a higher probability of receiving the respective outcome: In the gain condition, the advantageous symbol was associated with a 70% probability of winning 50 points and led to no outcome in only 30% of the trials in which it was chosen. The disadvantageous symbol was associated with a 30% probability of winning 50 points and led to no outcome in 70% of the trials in which it was chosen. Similarly, in the loss condition the advantageous symbol was associated with a 30% probability of losing 50 points, while the disadvantageous symbol had a loss probability of 70%. Additionally, we included a financially neutral control condition, which primarily served as a control condition for fMRI data analysis. Here, the two symbols likewise had a 70 and 30% probability of seeing neutral feedback, and no outcome otherwise. Symbols were assigned to the given conditions pseudorandomly. Trial order was randomized in 8 consecutive bins each comprising 30 trials (10 gain, 10 loss, 10 neutral). Within each bin these 30 trials were freely randomized. This was done to ensure a roughly equal number of trials per condition in each stage of the experiment.

**Fig. 1:**
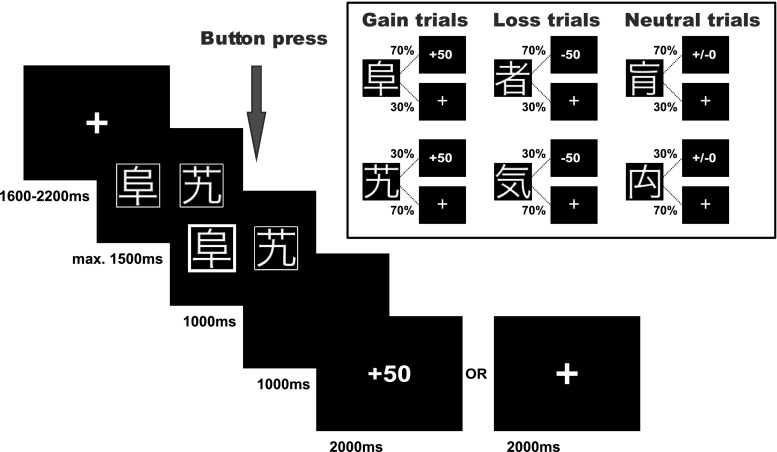
Trial structure, conditions, and cue-outcome contingencies of the probabilistic reinforcement learning task

Trial timing and conditions are displayed in Fig. 1. In short, the pair of symbols was presented for a maximum of 1500 ms and participants were asked to select one symbol. Once they had made their selection, the chosen option was highlighted for 1000 ms and a blank screen appeared during a 1000 ms delay period. Thereafter, the feedback occurred on the screen for 2000 ms. If the participants received no outcome a fixation cross was shown instead. If the participants did not press the button within 1500 ms after stimulus onset, the trial was aborted and the text “Too slow!” appeared on the screen. No response trials were omitted from all analyses (on average 2.55% of all trials).

Prior to the experiment, participants completed the Figurative Memory subtest from the Wechsler-Memory-Scale-R in order to evaluate the influence of (visual) working memory on learning performance that was previously reported in other studies (Collins and Frank 2012; Coppin et al. 2014). Participants were then instructed about the task and performed a practice run of 12 trials (four trials in each condition) outside the scanner. In the instructions, they were told that two stimuli would be presented in each trial and their task was to select one of them. Dependent on their choice they would win 50 points, lose 50 points, receive financially neutral feedback or no outcome. Participants were informed that the task comprised three conditions and that in each condition one cue had a higher probability of leading to an advantageous outcome. However, they did not know which cue was associated with a particular outcome. In addition, participants were informed that their net gain would be transformed into a monetary bonus at the end of the experiment. Upon completion of the experiment participants were interviewed about their retrospective task comprehension and knowledge about the cue-outcome contingencies. Finally, all participants were debriefed about the aim of the study.

### Ratings

Immediately before and after the learning experiment, we obtained subjective valence and arousal ratings for each symbol to determine changes in affective responses towards the stimuli. Here, each symbol was presented individually and rated according to valence and arousal on 9-point Self-Assessment Manikin visual analog scales (Bradley and Lang 1994). The sequence of stimulus presentations was pseudo-randomized.

### FMRI data acquisition

Functional and structural images were obtained using a 3T Siemens Trio MRI scanner. Functional images were acquired in a T2*-weighted blood oxygen level dependent sequence with a TR of 2000 ms, TE of 22 ms, flip angle of 90°, 64 × 64 in-plane matrix, field of view of 192 mm. Thirty-eight 2.5 mm slices with a 0.5 mm gap were measured in ascending order and 1098 volumes were acquired for the current study.

Additionally, a T1-weighted structural scan was recorded using a three dimensional MPRAGE sequence (matrix 256 × 240; 176 slices, FoV = 256 × 240 mm, voxel size = 1.0 × 1.0 × 1.0 mm, TR = 2300 ms, TE = 2.96 ms, flip angle = 9°) for participants that had not previously received a structural scan in our institute. For all other participants an existing T1-weighted structural scan showing similar imaging parameters was employed for co-registration.

A standard 12-channel head coil was used for the experiment. Visual stimuli were presented on a screen behind the scanner that was visible to the participants via a mirror mounted on the head coil.

### Behavioral data analyses

Statistical analyses of the behavioral performance data were carried out using IBM SPSS Statistics 20 (Armonk, NY, USA) with a level of significance being set at *p* < .05. For repeated-measures ANOVAs degrees of freedom were adjusted using Greenhouse-Geisser correction (Greenhouse and Geisser 1959) if the assumption of sphericity was violated. In this case, we report uncorrected degrees of freedom, corrected p-values and epsilon. For significant effects, generalized eta squared (η_G_^2^) as determined by R’s afex function is reported as a measure of effect size. Bonferroni-corrected t-tests were utilized as post-hoc tests where the ANOVA indicated a significant main or interaction effect. Cohen’s d is reported as a measure of effect size for independent samples t-tests.

### Computational learning model

Trial-wise PEs and participant- and condition-specific learning rates were derived from a reinforcement model. The model was previously applied in another implicit learning paradigm in healthy and clinical populations and it was shown to adequately capture probabilistic classification tasks (Mathar et al. 2017b). As a slightly modified version of standard Q-learning, our model contains separate learning rates for the experimental conditions that are fitted independently of other model parameters. The latter ensures that learning rates are statistically independent of the choice consistency parameter, which is not the case in standard Q-learning (Mathar et al. 2017b). More specifically, the reinforcement learning model consists of six input nodes ${I}_{i=1,...,6}$ with weighted connections to two output nodes (Q-values) ${Q}_{j=\text{1,2} }$that represent the presence or absence of the six different symbols (three pairs of symbols) and the two possible outcomes in each condition, respectively. On each trial, activity of the output nodes is computed as ${Q}_{j}= \sum _{i}{q}_{ij}{I}_{i},$ where ${q}_{ij}$is the weight connecting input node ${I}_{i}$ and output node ${ Q}_{j}$. Weights are initialized to 0.25, representing equal distribution of initial weights between the four connections that can be updated within one trial (connections from two input patterns to the two outcomes). Weights are updated in each trial by means of ${q}_{ij}(k+1)={{q}_{ij}\left(k\right)+\alpha }^{}{S}_{j}({R}_{j}-{Q}_{j}){I}_{i}$ where ${R}_{j}$ encodes the correct output in this trial, α constitutes a learning rate, and ${S}_{j}$ represents the subject’s response. The latter is included for allowing the model to simulate the behavior of the individual participant rather than optimal learning.

Since participants were informed about the three separate learning conditions and learning performance in one condition was independent of the other two conditions, we fitted three independent learning rates for the gain, loss, and neutral condition, respectively. This allowed us to differentially assess learning from reward (monetary gains) and punishment (monetary losses). For each participant, individual learning rates were determined that minimized the sum of squared differences between the model’s output and the participant’s response: $\sum _{jk}{\left({S}_{jk}-{Q}_{jk}\right)}^{2}$ → $min$, with $j=1, 2$ and $k$ being the number of trials. In a subsequent step, we modeled each participant’s choices of a particular outcome to follow a softmax distribution:

$$P\left( {choice={S_j}|{Q_1},{Q_2}} \right)=\frac{{{\text{exp}}\left( {\beta {Q_j}} \right)}}{{\exp \left( {\beta {Q_1}} \right)+{\text{exp}}\left( {\beta {Q_2}} \right)}}{\text{with}}\;j=1,2$$

with temperature or choice consistency parameter β. The parameter β was fitted to participants’ choices by minimizing the negative log likelihood of the choice probabilities P by $LL=-{ln}(\prod _{k}{P}_{k}\left({Q}_{j}\right))$, while the learning rates were held constant at the values optimized in the first step. As previously proposed by Mathar et al. (2017b), model fitting and estimation of all parameters was accomplished by nonlinear optimization.

### FMRI data analyses

MR images were preprocessed and analyzed using SPM8 (Wellcome Trust Centre for Neuroimaging, UCL, London, UK), implemented in Matlab 7.14 (The MathWorks Inc., Sherborn, MA). Functional images were unwarped and spatially aligned to the first image of the session to correct for movement artifacts. Realignment parameters were subsequently included as regressors of no interest in all individual participant level models described below. Slice timing correction to the anatomical middle slice was performed to correct for different acquisition times. The mean EPI image was co-registered to the high-resolution anatomical image, the T1 reference scan was segmented into different tissue classes, and functional and structural images were normalized to Montreal Neurological Institute (MNI) stereotaxic space. Subsequently, the normalized images were smoothed with an isotropic Gaussian kernel of 8 mm FWHM. The final resampled voxel size after normalization was 3 × 3 × 3 mm.

At the individual participant level, we set up separate models for the analyses of outcome-related BOLD responses, PE-related BOLD responses and functional connectivity: Stimulus- and outcome-related BOLD responses were modeled using three symbol pair regressors (gain condition trial, loss condition trial, neutral condition trial) and six outcome regressors (gain, gain omission, loss, loss avoidance, neutral outcome, no neutral outcome) that were modeled as impulse function and convolved with a hemodynamic response function. To examine outcome-related brain activation, individual contrast images for gain, gain omission, loss, and loss avoidance compared to neutral control trials were computed and submitted to separate one-sample t-tests for the analysis of within-group effects as well as two-sample t-tests for the comparison of outcome-related brain responses between lean participants and participants with obesity. For a detailed analyses of activation and deactivation patterns in contrasts revealing significant group differences, we extracted percent signal change of the BOLD signal using MarsBar 0.42.

PE-related brain activation was modeled at feedback onset and trial-wise PE estimates derived from the reinforcement learning model were used as parametric modulators of the feedback regressor that signaled the onset of any outcome in the gain or loss condition. Trials of the neutral condition were excluded from the analysis of PEs as performance was not reinforced by monetary feedback and participants may thus have been less attentive or motivated to learn the cue-outcome contingencies. Individual contrast images were submitted to one- and two-sample t-tests for within- and between group comparisons, respectively.

To investigate obesity-related changes in functional connectivity of the VS, we followed an approach proposed by Park et al. (2010) to build a psychophysiological interaction (PPI) term. Using this method, we examined the correlation of the observed BOLD time-series, without making assumptions about the neural event contributing to the BOLD signal (Kahnt et al. 2009). We focused on the VS as a seed region as previous studies have highlighted its importance in PE coding. First, we identified activated voxels in the left and right VS that significantly correlated with trialwise-PEs at whole group level. Here, anatomical ROI masks of the nucleus accumbens from the Harvard-Oxford Subcortical Structural Atlas were used to restrict the analysis. We then extracted individual participants time courses within the whole group activation masks, which were then multiplied by condition vectors that contained ones for four TRs after the presentation of positive (PPI regressor for positive PE feedback) and negative feedback (PPI regressor for negative PE feedback) and zeros otherwise. The resulting vectors were then used as regressors in an individual participant level model, which also included condition vectors containing separate feedback onsets for positive and negative feedback as well as realignment parameters as regressors of no interest. Contrast images of the PPI regressors were subsequently submitted to a 2nd level ANOVA comprising the factors PE condition (positive, negative) and group (lean, obese).

All results were corrected for multiple comparisons using a combination of individual voxel probability and cluster-extent based thresholds. Using 3dClustSim with an estimated non-Gaussian autocorrelation function and individual-voxel threshold of *p* < .001, we determined a cluster-extent based threshold of 53 adjacent voxels to reach a family-wise error rate of 5%.

### Association of neural responses and learning behavior

To examine if alterations in VS functional coupling are associated with learning success, we extracted for each participant individual beta weights for the functional connectivity between the VS and areas showing significant group differences (i.e. insula/superior temporal gyrus and vermis). These were then used as predictors of learning in a multivariate ANOVA including objective and subjective measures of learning success, namely the percentage of advantageous choices in gain and loss conditions during the acquisition phase, the average learning rate as well as subjective valence ratings.

#### Data availability

The datasets analyzed during the current study are available from the corresponding author on request.

## Results

### Behavioral performance

The net monetary outcome at the end of the experiment, percentage of advantageous choices and model-derived learning rates were evaluated as indices of individual learning performance. For the overall monetary score, an independent samples t-test revealed that individuals with obesity accumulated a significantly lower outcome than lean control participants over the course of the experiment [*t*(40) = 2.206, *p* = .037, *d* = 0.703].

**Fig. 2:**
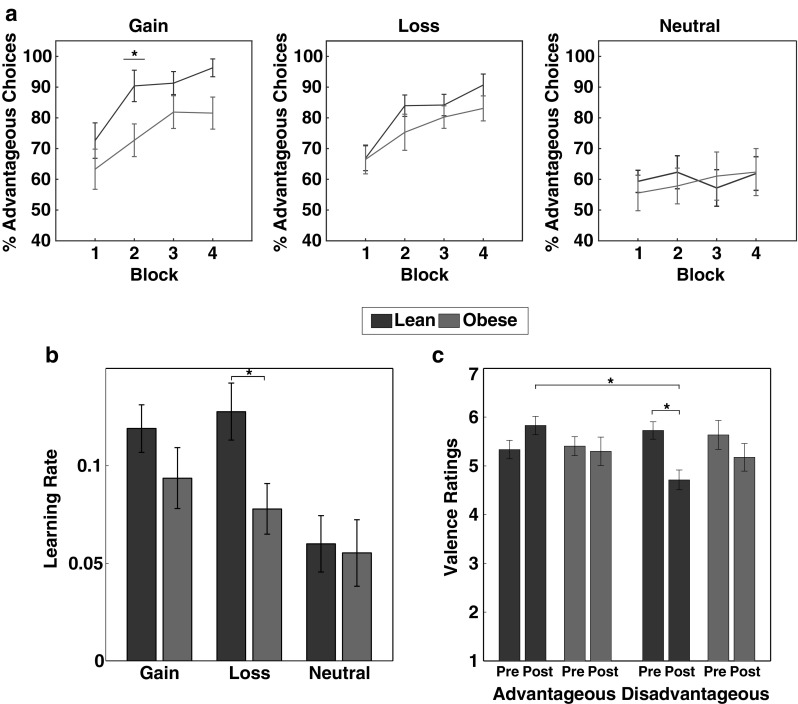
Behavioral results of the probabilistic reinforcement learning task. **a** Individuals with obesity showed a lower number of advantageous choices across gain and loss trials than lean participants during the acquisition phase (main effect of Group), which was particularly pronounced during the second block of the experiment (late acquisition phase, Group X Block interaction). No significant group differences occurred during the later phases of the experiment (blocks 3 and 4). Note that the neutral condition was not included in the statistical analysis, but is displayed here for completeness. **b** Individuals with obesity exhibited lower learning rates in the gain and loss condition than lean controls (main effect of Group). Again, the neutral condition was not included in the statistical analysis. **c** Valence rating obtained before and after the experiment revealed that lean participants showed a decrease in positive valence ratings for the disadvantageous choice options after the experiment as well as more positive ratings of advantageous compared to disadvantageous choice options after the experiment. No modulation of the subjective evaluation of the task stimuli occurred in individuals with obesity. Error bars represent standard errors of the mean taking into account the within-subject design (Cousineau 2005; Morey 2008). * *p* < .05 (two-tailed)

For the analyses of choice behavior, we calculated the percentage of advantageous choices in the gain and loss condition in 4 time bins (each comprising ~ 20 trials per condition). To evaluate learning performance, we then focused on choice behavior during the early phases of the experiment, when cue-outcome contingencies are predominantly acquired (e.g. Pessiglione et al. 2006; Lin et al. 2012; den Ouden et al. 2013). Specifically, we evaluated the percentage of advantageous choices during the first two blocks of the experiment. The neutral condition was excluded from this analysis since there was no financial incentive to develop a choice preference and participants may thus have used diverse behavioral strategies to complete the task (e.g. random choices or fixed choices of one symbol). Due to violations of normality, choice data were rank transformed and subjected to a repeated measures ANOVA including the within subject factors condition (gain, loss), block (1–2) as well as the between subject factor group (lean, obese). The results corroborate the previous finding: we found a significant main effect of group [*F*(1, 40) = 4.622, *p* = .038, η_G_^2^ = 0.049], a main effect of block showing an increase in correct responses from block 1 to block 2 [*F*(1, 40) = 50.560, *p* < .001, η_G_^2^ = 0.129], as well as a Group × Block interaction [*F*(1, 40) = 6.617, *p* = .014, η_G_^2^ = 0.019], indicating that individuals with obesity achieved a lower number of advantageous choices than lean controls particularly during the later acquisition phase (Block 2, *p* = .031; Fig. 2a). Interestingly, we found no significant modulation of learning performance by condition [main effect of Condition: *F*(1, 40) = 2.371, *p* = .131] and no significant interaction of group and condition, suggesting that group differences are comparable when learning from gain and loss feedback [interaction of Condition × Group: *F*(1, 40) = 1.671, *p* = .204].

Additionally, we examined choice behavior during the later phase (last two blocks) of the experiment, where the learning process should have resulted in stable cue-outcome associations. Here, we found no significant increase of performance across blocks [*F*(1, 40) = 3.984, *p* = .053] and no significant group differences across the gain and loss condition [main effect of group: *F*(1,40) = 1.259, *p* = .269; interaction of Condition × Group: *F*(1, 40) = 1.168, *p* = .286, Fig. 2a].

For the analysis of model-derived learning parameters, we extracted learning rates for the gain and loss condition separately and submitted them to a repeated-measures ANOVA including the within-subject factor condition (gain, loss) as well as the between-subject factor group (lean, obese). In line with the behavioral performance results, a significant main effect of group [*F*(1, 40) = 5.713, *p* = .022, η_G_^2^ = 0.076] indicates that lean participants exhibited significantly higher learning rates than individuals with obesity (Fig. 2b). As with the observed choice behavior, this effect was not modulated by the factor condition [interaction of Condition × Group: *F*(1, 40) = 0.839, *p* = .365].

In order to disseminate the influence of working memory on learning performance, we repeated the above-mentioned analyses including the Figurative Memory score as a covariate of interest. However, there was no evidence for a significant modulation of learning performance by individual working memory differences (Online Resource 1). Further, lean and obese participants did not significantly differ in their working memory performance (*U* = 167.500, *p* = .172).

### Ratings

To investigate differential changes in the evaluation of advantageous and disadvantageous symbols, we obtained individual valence and arousal ratings of all symbols before and after the experiment. Both were submitted to a repeated-measures MANOVA including the within-subject factors condition (gain, loss, neutral), time (before, after) and reinforcement probability (advantageous, disadvantageous) as well as the between-subject factor group (lean, obese). Using Pillai’s trace, we found a significant multivariate interaction effect of Time × Group × Reinforcement Probability [*V* = 0.157, *F*(2, 39) = 3.644, *p* = .035]. Univariate follow-up analysis showed that this effect was strongly driven by group differences in valence ratings [interaction of Time × Group × Reinforcement Probability: *F*(1, 40) = 4.635, *p* = .037, η_G_^2^ = 0.006]. Specifically, while participants with obesity exhibited similar ratings of advantageous and disadvantageous choice options before and after the experiment (all *p* > .05), lean participants showed a decrease in positive valence ratings for disadvantageous choice options from before to after the experiment (*p* = .045) as well as more positive valence ratings of advantageous compared to disadvantageous choice options after the experiment (*p* = .036; Fig. 2c).

Similar to objective markers of learning performance, we found no evidence for an association between the subjective evaluation of advantageous and disadvantageous symbols and figurative working memory (Online Resource 1).

### FMRI results

#### Gain receipt and loss avoidance

For the analysis of neural responses towards positive monetary outcomes, we first examined neural responses to monetary gains as well as responses to the successful avoidance of monetary losses, in each group individually. Subsequently, we compared lean and obese participants in a subtraction analysis.

Whole brain within-group analysis revealed that the receipt of a monetary gain was associated with significant activation in clusters encompassing the striatum, insula, anterior cingulate (ACC), middle frontal gyrus and midcingulate cortex (MCC) in lean and obese participants. Further, lean participants exhibited significantly higher activation to monetary gains than to neutral feedback in the right middle OFC, cerebellum and occipital cortex, while individuals with obesity showed increased activation in the inferior parietal lobule (Table 2).

**Table 2:** Within- and between-group comparison of whole-brain outcome processing results

Anatomical region	Cluster voxels	T at peak	Peak MNI coordinates
(a) Regions responding to gain receipt			
Lean			
Pallidum L	1676	6.58	− 12 5 − 5
*Insula L		6.07	− 33 14 − 11
*Insula R		5.87	36 17 − 8
Anterior cingulate cortex R	493	5.55	6 41 4
*Anterior cingulate cortex L		4.78	− 12 35 16
Inferior occipital gyrus R	330	4.84	39 − 88 − 11
*Cuneus R		4.58	15 − 100 7
*Vermis		4.15	3 − 64 − 38
Middle occipital gyrus L	239	4.83	− 15 − 103 4
*Lingual gyrus L		4.68	− 36 − 91 − 14
Middle orbitofrontal cortex R	87	4.81	33 59 − 5
Cerebellum L	136	4.45	− 27 − 58 − 29
Middle frontal gyrus R	108	4.27	45 47 19
Obese			
Anterior cingulate cortex L	979	5.60	0 35 16
*Medial orbitofrontal cortex R		4.93	3 41 − 5
Inferior parietal lobule R	78	5.49	57 − 37 52
Nucleus accumbens L	618	5.46	− 9 − 1 − 8
*Putamen R		5.04	15 14 − 5
*Putamen L		4.90	− 15 14 − 5
Midcingulate cortex R	128	5.29	0 − 13 31
Middle frontal gyrus R	54	4.52	39 41 19
Lean vs obese			
–	–	–	–
(b) Regions responding to loss avoidance			
Lean			
Insula R	171	6.01	33 23 − 5
Superior frontal gyrus R	101	5.16	30 62 − 2
Inferior parietal lobule R	199	4.75	45 − 49 43
*Angular gyrus R		4.34	42 − 61 52
Cerebellum L	82	4.39	− 12 − 76 − 29
Middle frontal gyrus R	59	4.31	42 11 52
Midcingulate cortex R	71	4.28	6 35 31
*Superior medial frontal gyrus R		3.78	6 29 43
Obese			
Inferior parietal lobule R	192	4.63	48 − 43 40
* Superior temporal gyrus R		3.35	63 − 49 19
Middle orbitofrontal cortex R	267	4.56	18 53 − 8
*Superior orbitofrontal cortex R		4.38	21 44 − 14
Middle frontal gyrus R	172	4.54	42 29 40
Cerebellum L	103	4.15	− 15 − 85 − 29
Lean vs obese			
–	–	–	–
(c) Regions responding to loss			
Lean			
Insula L	343	9.27	− 33 17 − 11
Insula R	391	8.64	33 20 − 8
Superior medial frontal gyrus R	643	6.03	3 35 40
*Anterior cingulate cortex R		5.73	9 35 22
Middle frontal gyrus R	484	5.54	24 56 22
*Middle orbitofrontal cortex R		4.74	36 56 − 8
Midcingulate cortex R	91	5.21	3 − 28 28
Midbrain L	502	5.11	− 3 − 13 − 11
*Midbrain R		4.96	3 − 19 − 17
*Thalamus L		4.90	− 9 − 10 − 2
Cerebellum L	91	4.80	− 15 − 76 − 32
Inferior parietal lobule R	166	4.76	42 − 52 43
Calcarine gyrus R	67	4.05	3 − 76 7
Obese			
Midcingulate cortex R	142	6.15	0 − 13 31
Anterior cingulate cortex R	1255	5.70	6 35 28
*Superior medial frontal gyrus R		5.56	6 41 34
Insula R	328	5.48	39 20 1
Inferior parietal lobule R	432	5.25	51 − 46 52
*Supramarginal gyrus R		4.86	57 − 49 31
Insula L	103	4.96	-30 17 − 11
Cerebellum L	99	4.70	− 12 − 82 − 26
Lean vs obese			
Medial prefrontal cortex L	61	-3.68	6 56 1
* Medial prefrontal cortex R		-3.56	− 9 53 1
(d) Regions responding to gain omission			
Lean			
Insula R	134	6.70	33 23 − 2
Insula L	60	5.27	− 27 20 − 2
Inferior parietal lobule R	97	4.37	45 − 49 43
Midcingulate cortex R	58	4.27	6 35 37
Obese			
Inferior parietal lobule R	75	3.85	51 − 49 49
Lean vs obese			
–	–	–	–

* Additional peak voxel in the current cluster

In both groups, the successful avoidance of monetary losses was similarly associated with higher activation in clusters encompassing the insula, middle frontal gyrus, cerebellum, and inferior parietal lobule. Additionally, lean participants demonstrated significant activation in the MCC, superior frontal, and superior medial frontal gyrus, whereas individuals with obesity showed increased activation in the middle OFC (Table 2).

The between-group analysis revealed that individuals with obesity and lean control participants did not significantly differ in their neural responses towards monetary gains or the successful avoidance of monetary losses.

#### Loss receipt and gain omission

In a second step, we examined neural responses following a negative monetary outcome. Specifically, we first analyzed the processing of monetary losses as well as the omission of monetary gains in each group individually. Subsequently, we compared responses of individuals with obesity and lean control participants in a between-group subtraction analysis.

In both groups monetary loss processing was associated with increased activation in clusters encompassing the insula, superior medial frontal gyrus, ACC and MCC, as well as cerebellum and inferior partial lobule. Additionally, lean participants displayed higher activation to monetary losses than neutral feedback in the thalamus, midbrain, and middle frontal gyrus (Table 2).

The omission of monetary gains elicited significant activation in the inferior parietal lobule in both groups. In lean control participants we further found activation in the insula, as well as MCC (Table 2).

**Fig. 3:**
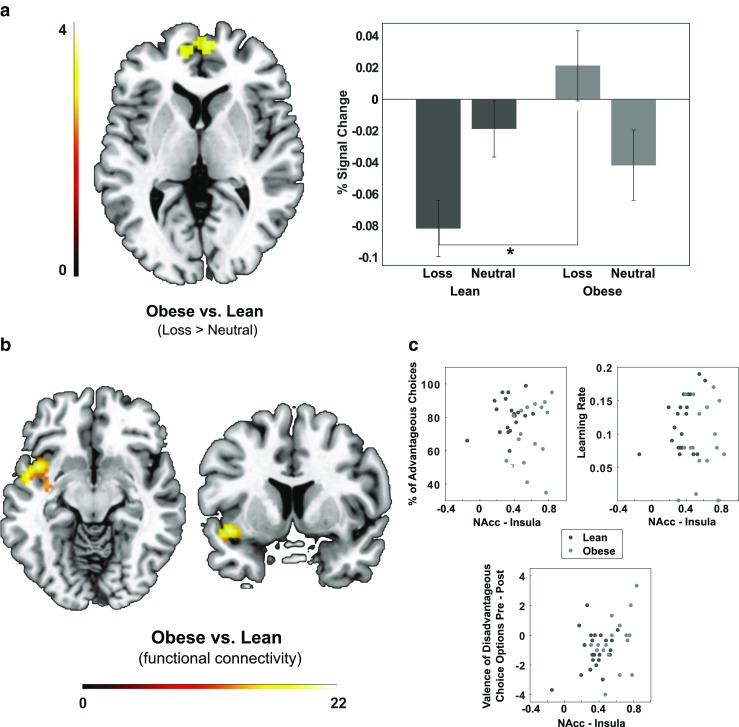
**a** Axial view *z* = 5 of the mPFC cluster (peak voxel at *x* = 6, *y* = 56, *z* = 1) showing significantly different activation in participants with obesity compared to lean participants during the processing of monetary losses compared to neutral outcomes. The graph additionally depicts the average percent BOLD signal change of this cluster for loss and neutral outcome trials in lean participants and individuals with obesity separately. Error bars represent standard error of the mean taking into account the within-subject design (Cousineau 2005; Morey 2008). **b** Axial and coronal views at *z* = − 10 and *y* = 8 demonstrating higher functional connectivity between the VS bilaterally and insula/superior temporal gyrus during PE processing in individuals with obesity compared to lean participants. **c** Connectivity strength was not significantly associated with indices of learning performance across all participants. * *p* < .05 (two-tailed)

The between-group analysis of monetary losses compared to neutral feedback revealed a region of significant differences in the medial prefrontal cortex (mPFC). Extracted percent signal change of the BOLD signal indicated that the effect was driven by significantly different neural responses to monetary losses [*t*(40) = 2.666, *p* = .013], such that lean participants demonstrated a pronounced deactivation in response to monetary losses, while individuals with obesity showed a small increase in activation (Fig. 3a).

#### PE representation

The within-group analysis of neural responses associated with PEs revealed that lean participants showed significant PE-related activity in the VS, and medial OFC, as well as superior temporal gyrus, occipital gyrus, MCC, and posterior cingulate gyrus. In individuals with obesity, PE-related activity occurred in the precentral gyrus, occipital gyrus and inferior parietal lobule (Table 3). Additionally, using a less conservative individual-voxel threshold (*p* < .005, 128 voxels) we likewise found evidence for significant PE-related activity in the VS bilaterally (*x* = 12, *y* = 8, *z* = − 11, *T* = 4.39; *x* = − 9, *y* = 14, *z* = − 11, *T* = 4.17).

**Table 3:** Within- and between-group comparisons of whole-brain prediction error processing results

Anatomical region	Cluster voxels	T at peak	Peak MNI coordinates
Lean			
Nucleus accumbens R	2224	8.27	12 2 − 8
*Nucleus accumbens L		8.20	− 12 5 − 11
*Medial orbitofrontal cortex L		7.26	− 6 47 − 8
Middle occipital gyrus L	801	7.33	− 18 − 97 − 5
*Cerebellum L		6.04	− 33 − 76 − 26
*Inferior occipital gyrus L		5.73	− 36 − 82 − 8
Superior temporal gyrus R	203	7.08	66 − 31 13
*Superior temporal gyrus R		4.27	66 − 19 4
*Postcentral gyrus R		4.07	66 − 10 25
Inferior occipital gyrus R	428	5.67	24 − 91 − 5
*Middle occipital gyrus R		4.82	27 − 97 4
*Middle occipital gyrus R		4.81	39 − 88 16
Superior temporal gyrus L	149	5.22	− 57 − 19 10
*Middle temporal gyrus L		4.87	− 57 − 31 7
Posterior cingulate cortex L	278	5.06	− 9 − 37 31
*Midcingulate cortex R		4.58	6 − 31 46
*Midcingulate cortex L		4.55	− 6 − 43 37
Obese			
Precentral gyrus R	200	6.64	30 − 13 37
*Postcentral gyrus R		6.48	30 − 25 43
*Superior frontal gyrus R		5.17	21 − 10 55
Inferior parietal lobule L	110	5.48	− 51 − 37 43
*Postcentral gyrus L		4.72	− 48 − 22 28
*Inferior parietal lobule L		4.54	− 33 − 43 34
Inferior occipital gyrus R	76	5.48	27 − 91 − 2
Lean vs obese			
–	–	–	–

* Additional peak voxel in the current cluster

The between-group comparison revealed that individuals with obesity and lean control participants did not significantly differ in PE-related activity (Table 3). To investigate the possibility that PE-related group differences occurred mainly during the acquisition phase, we additionally examined PE-related responses during the first two blocks of the experiment only. Again, we found no evidence for obesity-related alterations in neural PE representation (Online Resource 2).

#### VS functional connectivity

For the analysis of VS functional connectivity, the group-by-condition ANOVA indicated regions of significant group differences in clusters encompassing the left insula and superior temporal gyrus as well as between the VS and vermis/cerebellum (Table 4; Fig. 3b). Individuals with obesity compared to lean participants showed increased functional connectivity between the VS and these regions, while no modulation of group differences by condition (i.e. no significant Group × Condition interaction) was observed.

**Table 4:** Between-group comparison of ventral striatal functional connectivity during prediction error processing – main effect of group

Anatomical region	Cluster voxels	F at peak	Peak MNI coordinates
Superior temporal gyrus L	150	21.40	− 42 − 10 − 17
*Insula L		20.50	− 45 8 − 11
*Superior temporal gyrus L		19.17	− 51 2 − 11
Vermis	143	19.88	6 − 64 − 23
*Cerebellum R		14.38	9 − 49 − 17
*Cerebellum L		12.30	− 9 − 55 − 17

* Additional peak voxel in the current cluster

### Association of neural responses and learning behavior

Finally, we investigated the association of alterations in functional connectivity and learning behavior. Here, we used the strength of functional connectivity between the VS and the clusters showing significant group differences during outcome processing (insula/ superior temporal gyrus and vermis) to predict learning behavior of lean participants and participants with obesity. Surprisingly, we found no evidence for an association of learning success and connectivity strength. Using Pillai’s trace, there was no significant multivariate effect of VS connectivity with the insula/superior temporal gyrus [*V* = 0.077, *F*(3, 37) = 1.036, *p* = .388] or vermis [*V* = 0.166, *F*(3, 37) = 2.450, *p* = .079] on indices of learning (Fig. 3c). To rule out specific influences of connectivity on objective (learning rate, percentage of advantageous choices) compared to subjective measures of learning success (ratings), we further examined the univariate effects, but similarly found no evidence for any significant relationship.

## Discussion

In the current study, we aimed to investigate obesity-related alterations in non-food reinforcement processing, learning performance and the neural underpinnings of reinforcement-based learning in individuals with obesity. The results partly confirmed our hypotheses: (1) individuals with obesity compared to lean control participants showed alterations in the processing of monetary reinforcement stimuli. Specifically, we found differences during the processing of monetary losses, where lean participants responded with a strong deactivation, while individuals with obesity exhibited a small increase in activation of the mPFC. Contrary to our hypothesis, we found comparable activation patterns in reward-related areas in both groups for the processing of monetary gains. (2) In line with previous studies, individuals with obesity exhibited a compromised learning performance. This was evidenced by a lower number of advantageous choices as well as lower learning rates in individuals with obesity. In the same vein, subjective indices of reinforcement-based learning suggested that lean, but not obese, participants’ evaluation of the task stimuli was modulated by learning experience. (3) Lastly, both groups showed similar neural PE representations in the VS, but individuals with obesity exhibited higher functional connectivity following feedback between the VS and a cluster encompassing the insula and superior temporal gyrus. This was, however, not predictive of a compromised learning performance in individuals with obesity.

### Outcome processing

In the current study, we provide further evidence for generalized obesity-related alterations in reinforcement processing beyond the food context. We found evidence for aberrant neural responses of the mPFC after actual monetary losses in individuals with obesity, which indicate that the processing of negative reinforcement may be associated with altered value representations in obesity.

To date little evidence exists on the processing of negative events in individuals with obesity. Opel et al. (2015) found obesity-related differences in the coding of monetary rewards and no differences in the coding of punishment, but used relatively higher gains than losses. Employing comparable gains and losses, Balodis et al. (2013) reported obesity-related differences in the neural representation of anticipated and received monetary losses. They found that the presentation of an early predictive cue indicating an upcoming monetary loss was associated with relatively higher neural responses to anticipated losses than neutral monetary outcomes in areas of the brain’s reward circuit, while actual monetary losses compared to financially neutral feedback lead to relatively decreased medial frontal activation in participants with obesity. In our study, we found an obesity-related modulation of mPFC activation for monetary losses compared to neutral feedback. Importantly, however, a separate inspection of activation and deactivation patterns towards monetary losses and financially neutral outcomes revealed that this was driven by a slight increase in response to monetary losses in individuals with obesity, which stood in contrast to a pronounced deactivation in lean control participants.

The mPFC, in particular its ventral subdivision, has been hypothesized to provide a common valuation system for different reinforcers, showing greater BOLD responses to more rewarding or less aversive stimuli (Bartra et al. 2013). This has been reported for money and food (Levy and Glimcher 2011; Sescousse et al. 2013) as well as the encoding of the emotional value of pictures (Winecoff et al. 2013). These neural responses are often characterized by opposing patterns of activity with higher activation to the presentation of more rewarding and deactivation to more negative (Winecoff et al. 2013) or less valuable stimuli (Mullett and Tunney 2013). Canessa et al. (2013) reported that alterations in the activation patterns of the brain’s reward circuit may be associated with behavioral responses towards potential losses, such that larger loss-related deactivation than gain-related activation predict higher loss aversion during decision making. Indeed, Tom et al. (2007) found that greater neural sensitivity to increasing losses in the medial OFC, insula, and striatum were associated with greater behavioral loss aversion in a gambling paradigm, supporting the notion that individual differences in cortical sensitivity to aversive stimuli affect cognitive performance and decision making.

It has been suggested that obesity is characterized by a two-fold pattern of reward responses encompassing heightened anticipatory, but blunted consummatory neural responses to rewarding stimuli (Kenny 2011). Previous studies in the context of monetary reward have already shown mixed results with increased anticipatory (Balodis et al. 2013), but both increased (Opel et al. 2015) and decreased (Balodis et al. 2013) consummatory responses to monetary gains. Though the design of the current study was focused on outcome processing and did not allow for a thorough investigation of anticipatory processes, we find evidence for a decreased responsiveness to the receipt of negative stimuli in obesity. Surprisingly, we do not find differences in the neural processing of monetary gains, suggesting that reward processing may not be universally altered in individuals with obesity and differences in task design need to be considered.

In conclusion, our results indicate that individuals with obesity exhibit aberrant value representations of monetary losses in the mPFC. A decreased motivational significance of negative action consequences could be an integral mechanism contributing to alterations in decision making, such as a preference for immediate rewards in the light of long-term negative consequences (Horstmann et al. 2011) or a higher valuation of temporally close, but objectively worse decision outcomes (Simmank et al. 2015). Similarly, whether individuals with obesity will change or maintain their eating behavior can be strongly determined by their perception of its consequences. As evidence suggests that these mechanisms may be generalized across different domains of reinforcement, a lower motivational significance of negative (health) consequences of overeating may thus potentially decrease their regulatory effect on eating behavior, facilitating maintained dysfunctional eating patterns even in the light of negative long-term consequences.

### Learning performance

In addition to non-food incentive representation, we also evaluated group differences in reinforcement-based learning performance. Similar to previous studies (Coppin et al. 2014; Horstmann et al. 2011), we found evidence for a lowered reinforcement-based learning performance in individuals with obesity. Interestingly, data on the subjective evaluation of the presented stimuli, as indicated by valence ratings, suggested that this effect was driven by alterations in differential conditioning, such that differences were particularly pronounced for the evaluation of the disadvantageous stimuli across conditions. While lean participants evaluated the disadvantageous stimuli as less pleasant after the experiment and showed a clear differentiation in valence ratings between advantageous and disadvantageous symbols, individuals with obesity demonstrated no modulation of their ratings by learning experience. This is in line with previous studies that similarly showed obesity-related impairments particularly when learning the meaning of cues that have a low probability for subsequent rewards. Specifically, Zhang et al. (2014) reported that women with and without obesity responded comparably towards the cues that were associated with a food reward, but women with obesity showed higher reward expectancies towards the other cue that was in fact never followed by a food reward. In the same vein, Coppin et al. (2014) found that individuals with obesity were particularly impaired in avoiding disadvantageous options in a probabilistic learning task. In an earlier study from our group, we used the Weather Prediction Task to investigate PE coding in individuals with obesity in a complex implicit learning task. Adding to previous results, we found selective impairments on the neural level, namely in the utilization of negative feedback and PEs for learning in individuals with obesity (Mathar et al. 2017a). Interestingly, rodents studies point in a similar direction showing that rats fed on highly palatable cafeteria diets are insensitive to aversive stimuli, i.e. they do not decrease food consumption in the light of a conditioned stimulus that is predictive of a aversive foot shock (Velazquez-Sanchez et al. 2015), an effect that may be mediated by alterations in the striatal D2 receptor system (Johnson and Kenny 2010). This deficit seems to be selective to negative stimuli, as rats fed on Western diets fail to solve tasks, in which a (negative) feature stimulus signals that a subsequent conditioned stimulus will not be paired with an expected reward, while they are not impaired in similar tasks using positive feature stimuli (Kanoski and Davidson 2011). Together, previous studies in humans and animals point at obesity-related alterations in negative outcome learning.

Here, we extended this work by applying a task that explicitly separates effects of learning from monetary gains (and their omission) versus learning from monetary losses (and their successful avoidance). Interestingly, none of the learning indices displayed condition effects, suggesting that learning performance is not primarily related to the actual monetary value of the presented outcomes. Rather it depends on their relative meaning discriminating disadvantageous from advantageous choice options.

### PE processing and functional connectivity

A lower reinforcement-based learning performance has been shown to relate to alterations in the neural representation of dopaminergic learning signals in the striatum (Schönberg et al. 2007; Park et al. 2010; Eppinger et al. 2013). In the current study, individuals with obesity showed no alterations in the regional PE coding *per se*, but exhibited significantly higher functional connectivity between the VS and a cluster encompassing the left insula, and superior temporal gyrus during the processing of monetary outcomes. However, as opposed to other studies, this was in fact not directly related to decreases in learning performance, suggesting that alterations in VS-insula connectivity may rather reflect more general changes in the processing of (unexpected) feedback than differences in the utilization of striatal signals for learning.

The insula is a key area for the processing of interoceptive sensations and a node for the integration of external and interoceptive inputs (Craig 2002, 2009, 2011; Critchley et al. 2004). Predominantly the (ventral) anterior insula seems to be related to affective processing and autonomic function (Kelly et al. 2012; Chang et al. 2013). Interestingly, VS and insula are anatomically connected (Leong et al. 2016) and commonly co-activate in task-based and resting state fMRI studies (Postuma and Dagher 2006; Cauda et al. 2011; Chang et al. 2013). Evidence suggests bidirectional connectivity patterns between (anterior) insula and VS during incentive processing. More precisely, the insula has been hypothesized to code somatic changes in response to appetitive and aversive stimuli and project to the VS to facilitate motivated behavior (Clithero et al. 2011; Cho et al. 2013). Furthermore, a higher tract coherence between the anterior insula and NAcc has been shown to be negatively related to risk preferences (Leong et al. 2016). Likewise, the VS has been found to project to the insula particularly during high attention allocation to appetitive cues (Rothkirch et al. 2014).

Combined, these results highlight the possibility that an increased connectivity of insula and VS in individuals with obesity may reflect a stronger engagement of the reinforcement processing circuitry and increased attention allocation in response to the presentation of monetary feedback. However, this does not directly translate to learning performance, suggesting that potential differences in affective coding do not impact *per se* on reinforcement-based learning performance in individuals with obesity.

### Other mechanisms in reinforcement-based learning

Other mechanisms may contribute to obesity-related alterations in reinforcement-based learning, instead. Indeed, learning and complex choice behavior have been discussed to rely on a combination of mechanisms beyond simple model-free learning based on striatal PEs only (Collins and Frank 2012; Doll et al. 2016). For instance, working memory capacity may play a distinct role in associative learning, particularly for so-called model-based learning processes that rely on building mental representations of the task environment. In complex 2-step learning tasks, designed to investigate such model-based compared to model-free processes, Parkinson patients with higher working memory capacity have been found to exhibit more model-based decisions (Sharp et al. 2016). Moreover, individuals were shown to be more resilient against the disruption of performance by external factors (Otto et al. 2013; Smittenaar et al. 2013). Similarly, Collins and Frank (2012) found that the combination of simple reinforcement-based learning models with working memory capacity best explained participants’ behavior in a putatively simpler instrumental learning task. For individuals with obesity, Coppin et al. (2014) reported working memory impairments and suggest that this may contribute to their failure to form preferences for highly rewarded stimuli. It is thus plausible to assume that working memory capacity contributed to learning deficits in the current study, though, surprisingly, we did not find a significant association in the data. This may be due to methodological issues: Firstly, we employed a simple working memory task in which both groups performed very well and performance variance was relatively small. Secondly, we focused on visual working memory, while other studies have employed different measures. This may suggest that performance in the current task did not depend on the ability to memorize complex visual stimuli, but leaves the possibility that other and more sensitive measures of working memory capacity may help to further elucidate potential mechanisms contributing to obesity-related alterations in reinforcement-based learning.

### Strength, future directions and limitations

To our knowledge, the current study is the first fMRI study integrating behavioral as well as neural correlates of monetary reinforcement processing and reinforcement-based learning in individuals with obesity. While previous studies have mostly focused on general correlates of learning and response adaptation, the current paradigm allows for the investigation of two additional aspects: (1) a clear separation of learning from monetary gains compared to losses, and (2) the examination of both objective markers of learning performance and the subjective evaluation of the conditioned stimuli.

However, we could not conclusively resolve which underlying mechanisms contributed to obesity-related learning alterations in the current study. Thus some further aspects should be considered in future studies. Firstly, a relatively low overall sample size precluded the examination of gender differences in the current task, though previous studies have shown that alterations in executive functioning and behavioral adaptation may be particularly pronounced in women with obesity (Horstmann et al. 2011; Zhang et al. 2014). In the same vein, overweight participants should be included in future studies, as overweight and moderately obese participants seem to be more distinct from lean participants in reward sensitivity, working memory performance and monetary reward processing than individuals with severe obesity (Davis et al. 2004; Coppin et al. 2014; Dietrich et al. 2014; Verdejo-Román et al. 2017).

Additionally, while learning mostly took place during the first half of the experiment, performance in the second half was likely more influenced by fatigue and individual tendencies to exploit the learned associations or explore other options despite existing knowledge of the advantageous choice options. More dynamic paradigms with changing cue-outcome contingencies could reduce these potential biases.

Lastly, the current study was mainly focused on the utilization of feedback for learning. However, in order to understand the influence of altered negative value representations on behavior in individuals with obesity, additional measures of decision making and the processing of negative action outcomes, e.g. in the context of eating behavior and health consequences, should be employed in future studies.

## Conclusion

The current study examined the neural representation of non-food reinforcement stimuli and their utilization for reinforcement-based learning in individuals with obesity employing a probabilistic learning paradigm with separate monetary gain and loss learning conditions. Findings of aberrant negative value representations and increased functional connectivity between the VS and insula point at generalized obesity-related differences in neural reinforcement processing that are present outside of the food context. Additionally, a reduction in reinforcement-based learning performance and specific alterations in disadvantageous outcome learning further support the idea of a lower impact of negative choice consequences on behavioral adaptation in individuals with obesity. Surprisingly, neither PE-related processes nor working memory explained obesity-related differences in learning, highlighting the need for further investigations, with potentially different methodological approaches.

## Electronic supplementary material

Below is the link to the electronic supplementary material.

Statistical results showing no significant influence of working memory on subjective and objective markers of learning performance. (PDF 136 KB)

Within-group and between-group fMRI results on PE processing in individuals with obesity and control participant during the first two blocks of the experiment (acquisition phase). (PDF 75 KB)
