# Supplementary material for: Altered monetary loss processing and reinforcement-based learning in individuals with obesity
Source: Brain Imaging Behav. 2017 Dec 29;12(5):1431–49. doi: 10.1007/s11682-017-9786-8 (PMC6290732; doi:10.1007/s11682-017-9786-8)
Supplement: Supplementary file 1 — Statistical results showing no significant influence of working memory on subjective and objective markers of learning performance. (PDF 136 KB) [file 11682_2017_9786_MOESM1_ESM.pdf]

Online Resource 2

Supplementary Table II.

Within- and between-group comparison of whole-brain prediction error processing results during the first two blocks of the experiment.

Anatomical region	Cluster voxels	T at peak	Peak MNI coordinates
**Lean**			
Nucleus accumbens R	4926	7.72	15 8 -8
*Amygdala L		7.26	-18 -1 -8
Postcentral gyrus L	116	4.33	-57 -16 16
*Heschl gyrus L		4.30	-63 -10 10
*Superior temporal gyrus L		4.12	-60 -31 22
Midcingulate cortex R	81	4.32	3 -22 49
*Midcingulate cortex L		4.15	-3 -34 46
**Obese**			
Supramarginal gyrus R	115	6.35	66 -22 40
*Middle frontal gyrus R		5.01	48 -4 52
*Postcentral gyrus R		4.20	57 -13 49
Precentral gyrus L	229	5.53	-33 -16 49
*Inferior parietal lobule L		4.97	-54 -37 46
*Precentral gyrus R		4.96	36 -19 40
Olfactory cortex R	83	5.11	3 14 -5
*Nucleus accumbens R		4.71	12 11 -8
*Nucleus accumbens L		4.52	-6 8 -8
Supramarginal gyrus L	79	4.56	-48 -31 25
**Lean vs. Obese**			
---	---	---	---

Kube et al. Altered monetary loss processing and reinforcement-based learning in individuals with obesity.

Corresponding author: Jana Kube, kube@cbs.mpg.de, Max Planck Institute for Human Cognitive and Brain Sciences, Leipzig; Leipzig University Medical Center, IFB AdiposityDiseases
