# Supplementary material for: Altered monetary loss processing and reinforcement-based learning in individuals with obesity
Source: Brain Imaging Behav. 2017 Dec 29;12(5):1431–49. doi: 10.1007/s11682-017-9786-8 (PMC6290732; doi:10.1007/s11682-017-9786-8)
Supplement: Supplementary file 2 — Within-group and between-group fMRI results on PE processing in individuals with obesity and control participant during the first two blocks of the experiment (acquisition phase). (PDF 75 KB) [file 11682_2017_9786_MOESM2_ESM.pdf]

Online Resource 1

Supplementary Table I

Statistical results of the univariate and repeated measures ANCOVAs examining the influence of working memory on learning performance.

Learning Performance	ANCOVA main effect of WMS-R FM	
	F	*p*
Score	0.018	.895
Advantageous choices	0.572	.454
Learning Rate	3.374	.074
Ratings		
*Valence	0.937	.339
*Arousal	2.252	.141

Note. WMS-R FM – Wechsler Memory Scale – Revised, Subtest Figural Memory. F and p values for the main effect of working memory score on parameters of learning performance are reported here.

Kube et al. Altered monetary loss processing and reinforcement-based learning in individuals with obesity.

Corresponding author: Jana Kube, kube@cbs.mpg.de, Max Planck Institute for Human Cognitive and Brain Sciences, Leipzig; Leipzig University Medical Center, IFB AdiposityDiseases
